# Contribution of reformulation, product renewal, and changes in consumer behavior to the reduction of salt intakes in the UK population between 2008/2009 and 2016/2017

**DOI:** 10.1093/ajcn/nqab130

**Published:** 2021-05-08

**Authors:** Mathilde Gressier, Franco Sassi, Gary Frost

**Affiliations:** Section for Nutrition Research, Department of Metabolism, Digestion and Reproduction, Faculty of Medicine, Imperial College London, London, United Kingdom; Centre for Health Economics & Policy Innovation, Department of Economics & Public Policy, Imperial College Business School, Imperial College London, London, United Kingdom; Centre for Health Economics & Policy Innovation, Department of Economics & Public Policy, Imperial College Business School, Imperial College London, London, United Kingdom; Section for Nutrition Research, Department of Metabolism, Digestion and Reproduction, Faculty of Medicine, Imperial College London, London, United Kingdom

**Keywords:** reformulation, public health, food policies, intervention evaluation, consumer behavior, sodium, salt

## Abstract

**Background:**

The UK salt reduction program started in 2003, consisting of education campaigns to raise awareness about the risks associated with a high-salt diet and of a reformulation strategy for food manufacturers. This program is often cited as an example of a successful public health program.

**Objectives:**

This study aimed to assess: *1*) the impacts of changes in food composition and changes in consumer behavior on sodium intakes; and *2*) whether changes were similar across socioeconomic groups.

**Methods:**

Food intakes for the UK population were derived from food diaries in the UK National Diet and Nutrition Survey for 2008/09 (year 1; *n* = 1334) and 2016/17 (year 9; *n* = 995). Year-specific sodium densities of foods were used to calculate the average sodium density of all food and beverage consumed. Changes in sodium density between the 2 years were explained by changes in food composition (change in sodium density of products) and/or changes in behavior (type and quantity of food consumed) using a decomposition approach.

**Results:**

The program was linked to a 16% (95% CI: −21% to −12%) decrease in sodium intake between years 1 and 9, while the sodium density of foods consumed decreased by 17% (95% CI: −21% to −12%). This decrease was largely driven by reformulation (−12.0 mg/100 g). Changes in food choices reinforced the effects of the program, but had a smaller impact (−1.6 mg/100 g). These effects were similar across socioeconomic groups, whether stratified by education or income, with a consistent effect of reformulation across groups and no differences between groups in behavioral responses to the program.

**Conclusions:**

A multi-component sodium reduction strategy deployed in the United Kingdom starting in 2003 corresponded to an important reduction in sodium intakes for the population. This reduction was mostly driven by changes in the food environment (reformulated food products to reduce the sodium density of foods) and, to a smaller extent, by changes in food choices. Impacts were consistent across socioeconomic groups.

## Introduction

High blood pressure is the third leading risk factor for premature death in the United Kingdom, affecting more than 25% of adults (1). It is estimated to cost £2.1 billion every year for the UK National Health Services (2). Contributors to high blood pressure risk include both nonmodifiable (e.g., genes, age) and modifiable factors (2). A leading contributor among the latter is high dietary sodium intake (3). In 2005/2006, the mean salt intake was 8.8 g/d in the UK population, well above the 6 g/d national recommendation (2). As a strategy to reduce the prevalence of high blood pressure, the WHO called on governments to act on reducing salt intakes (4). The UK government was among the first to launch a salt reduction campaign, in 2003 (5). The campaign had 2 objectives: incentivizing food manufacturers to reduce salt in their foods (with category-specific reformulation targets) and educating the population about how to reduce salt intake. The salt reduction campaign is still active.

Evidence suggests that salt intakes in the UK population decreased by 11% between 2005/2006 and 2014 (2). The factors driving this decrease are not yet fully understood. Previous research has shown that approximately two-thirds of the population were aware of the salt guidelines in 2007 (6), and that manufacturers reduced the sodium contents of their foods (7). The decrease in salt intakes was found to be larger during the initial phase of the salt reduction campaign (from 2004 to 2010) than during the second phase (from 2011) (8). Applying an original method that decomposed the change in salt purchases between the effects of reformulation, consumer behavior, and product renewal, Griffith and colleagues (9) found that the reduction in sodium from foods bought in supermarkets came mainly from reformulation, and not from changes in consumer behavior. However, this study only took into consideration food purchased for at-home consumption.

To improve our understanding of the pathways through which the UK salt reduction campaign succeeded in reducing salt intakes, we investigated the effects of changes in the demand for and supply of the food products contributing to sodium intakes in the United Kingdom. Building on the method developed by Griffith et al. (9), the change in sodium density of all foods consumed by the population in 2008/09 and 2016/17 was decomposed between changes in the sodium density of food products (i.e., reformulation and change in composition due to the renewal of products), and changes in food choices. Changes in food choices between and within food categories were unraveled to further understand how food choices impacted salt intakes.

## Methods

### Data source

We derived population estimates of dietary intakes from the National Diet and Nutrition Survey (NDNS) rolling program (10). We used surveys from 2008/09 (Year 1) to 2016/17 (Year 9). For each survey year, data were collected from a sample of approximately 1000 individuals representative of the UK population. Participants were asked to fill out a 4-d food diary by listing all foods and beverages consumed during the day. Records from participants who filled out information from 3 or 4 d were included in the analysis. We selected individuals with valid measures of BMI, self-declared ethnicity, and income (and education for adults; **Supplemental Figure 1**). The validity of the diary was tested by assessing energy in relation to biomarkers (doubly labeled water) (11), but there was no correction for underreporting. Correction for underreporting is usually done by calculating the ratio between estimated energy expenditure and measured energy intakes. As estimations of energy expenditure obtained through the NDNS are already subject to bias, we did not feel it was appropriate to adjust for energy, as this could have further biased the results (11, 12). Plus, as we were examining changes over time, this would not affect the results if we assume the bias is similar at different time points. Year-specific nutrient information was taken from the UK Nutrient Databank, and foods’ nutrition compositions were linked to food codes corresponding to food items reported by participants. Similar branded products matching the same definition of foods consumed by participants were aggregated into the same NDNS food code. Information on discretionary salt added while cooking or while eating was not expressly requested, but some participants voluntarily declared when they were adding salt in recipes or while eating. In addition to the food survey, some participants had other health measurements performed, such as a blood pressure measurement or a 24-h urinary sodium excretion measurement, in survey years 1 to 5. The estimation of sodium intakes using 24-h urine collection is more reliable than using food surveys; however, 24-h urine collection was only performed on a subset of participants. Also, this measurement was not performed after year 5, as the UK sodium survey, to monitor the UK population sodium intakes, was done separately from the NDNS rolling program (13).

To analyze the main food sources of sodium, and how they changed over time, we classified the NDNS food codes into the food categories from the McCance and Widdowson’s table (14). These food categories gather foods with common characteristics, such as cereals and cereal products, milk and milk products, or vegetables. We used the NDNS Main Food Group classification to create 13 food categories.

### Outcome measures

The primary outcome was the change in mean sodium density of food eaten by the population, and its decomposition into the effects of product reformulation, product renewal, and consumer switches. The mean sodium densities (and SEMs) of foods eaten by the UK population were calculated in year 1 and year 9. To obtain population mean daily food intakes, we first averaged quantities eaten by each individual over the 3 or 4 d of the survey to have individuals’ usual intakes. We calculated the proportion (in weight) of each food consumed for each participant. Then, from all individuals’ usual intakes, we calculated the average proportion of each food code eaten by the population, using survey weights.

The mean sodium density over all foods consumed by the population in a year (${N_t}$) was obtained by weighting the proportion of each food code (i) eaten by its sodium density at year t (${n_{{\rm{i,t}}}};$**Equation 1**).

(1) $$\begin{eqnarray*}
{\rm{\ }}{N_{\rm{t}}} = \mathop \sum \limits_i {w_{i{\rm{t}}}}{n_{{\rm{i}},{\rm{t}}}}\ \hbox{, with }{w_{i,t}} = \frac{{weight{\rm{\ }}food{\rm{\ }}i}}{{weight{\rm{\ }}all{\rm{\ }}foods}}\
\end{eqnarray*}$$

As sodium density varies greatly between solid and liquid foods, we estimated both sodium density for all foods and beverages consumed and for solid foods only. In addition, because of the possibly biased estimation of discretionary salt added while cooking or eating, we calculated the sodium density of solid foods when excluding discretionary salt (“table salt”).

Secondary outcomes studied were daily sodium intakes and blood pressure, also obtained from the NDNS survey. These measurements were taken from subsamples of participants who had completed food diaries. Daily sodium intakes were obtained from the food diaries for years 1 to 9, as well as from urinary measures of salt excretion from year 1 to 5 of the NDNS. Urine was collected over 24 h to get an accurate picture of sodium ingested and excreted during a day. Quality control procedures were done to measure the validity of the 24-h urine sodium excretion (15). Measures of blood pressure were obtained by averaging the last 2 of 3 measurements by the nurse. Numbers of participants included in the measurements of urinary salt excretion and blood pressure are shown in **Supplemental Figures 2** and **3**.

### Decomposition of sodium density (statistical analysis)

Changes in the sodium density between year 1 and year 9 were decomposed between the effects of reformulation, product renewal, and switches between products, using the method created by Griffith and colleagues (9) and given in **Equation 2**. The product renewal effect measures the difference between the sodium consumed from products exiting the survey (i.e., not consumed anymore) and products entering the survey (i.e., new products consumed). Food codes are separated into 3 groups: continuous food codes consumed in year 1 and year 9 (group C), new food codes only present in year 9 (group N), and exiting food codes only present in year 1 (group X).

(2) $$\begin{eqnarray*}
\Delta {N_t} &=& \mathop \sum \limits_{i \in C}^{} {w_{i,{t_0}}}\left( {{n_{i,{t_1}}} - {n_{i,{t_0}}}} \right) + \mathop \sum \limits_{i \in C}^{} ({w_{i,{t_1}}} - {w_{i,{t_0}}})\left( {{n_{i,{t_1}}} - {n_{i,{t_0}}}} \right) \\
&& + \mathop \sum \limits_{i \in C}^{} ({w_{i,{t_1}}} - {w_{i,{t_0}}})\left( {{n_{i,{t_0}}} - {N_{{t_0}}}} \right) + \mathop \sum \limits_{i \in N}^{} {w_{i,{t_1}}}\left( {{n_{i,{t_1}}} - {N_{{t_0}}}} \right)\\
&& - \mathop \sum \limits_{i \in X}^{} {w_{i,{t_0}}}({n_{i,{t_0}}} - {N_{{t_0}}})
\end{eqnarray*}$$

The first sum on C (continuous) reflects reformulation—the effect of a change in the sodium density of a food—keeping quantities consumed constant. The second and third sums on C reflect population switches between products (products than were or were not reformulated), whiles the sums on N (new) and on X (exiting) reflect the effects of product renewal.

The decomposition was firstly performed to evaluate the changes in the average sodium density of foods consumed by the UK population, considering either all foods and beverages consumed, only solid foods, or solid foods except table salt.

Secondly, we decomposed the change in daily sodium density, aggregated at the food category level. The switching effect was split to differentiate switches within a category and between categories (**Equation 3**). This was done by adding and subtracting the nutrient density of the food category *k* (${N_{k,\ {t_0}}})\ $relative to the weight of this category (${W_k}$), as shown in Equation 3. The term in $( {{n_{i,\ {t_0}}} - \frac{{{N_{k,\ {t_0}}}}}{{{W_k}}}} )$ indicates the effects of switches compared to the density of the specific category: that is, switches within a category. The term in $( {{N_{{t_0}}} - \frac{{{N_{k,\ {t_0}}}}}{{{W_k}}}} )$ indicates the effects of switches from a category compared to the density of all foods: that is, changes between categories.

(3) $$\begin{eqnarray*}
\Delta {N_{k,t}} \!&=&\! \mathop \sum \limits_{i \in k \cap C}^{} {w_{i,{t_0}}}\left( {{n_{i,{t_1}}} \!-\! {n_{i,{t_0}}}} \right) + \mathop \sum \limits_{i \in k \cap C}^{} ({w_{i,{t_1}}} \!-\! {w_{i,{t_0}}})\left( {{n_{i,{t_1}}} \!-\! {n_{i,{t_0}}}} \right)\\
&& + \mathop \sum \limits_{i \in k \cap C}^{} ({w_{i,{t_1}}} - {w_{i,{t_0}}})\left( {{n_{i,{t_0}}} - \frac{{{N_{k,{t_0}}}}}{{{W_k}}}} \right) - \left( {{N_{{t_0}}} - \frac{{{N_{k,{t_0}}}}}{{{W_k}}}} \right)\\
&&\times \mathop \sum \limits_{i \in k \cap C}^{} ({w_{i,{t_1}}} - {w_{i,{t_0}}})+ \mathop \sum \limits_{i \in k \cap N}^{} {w_{i,{t_1}}}\left( {{n_{i,{t_1}}} - {N_{{t_0}}}} \right)\\
&& - \mathop \sum \limits_{i \in k \cap X}^{} {w_{i,{t_0}}}({n_{i,{t_0}}} - {N_{{t_0}}})
\end{eqnarray*}$$

Thirdly, we applied the decomposition for each food category taken individually. In that case, we adapted Equation 2 with the food product weights rescaled at the food category level (such that the sum of all food product weights from the same category equaled 1). This allowed us to estimate the intensity of reformulation in each category (**Supplemental Equation*1***).

The decomposition was further applied on subgroups of the population, defined by demographic characteristics (age group, sex) or socioeconomic status (SES; equivalized income, higher qualification achieved for adults). The definitions of continuous, new, and exiting products (groups C, N and X) were not adjusted to the recorded consumption by subgroup of the population. We considered that as long as the product was recorded by an individual in the survey, it was available for the population (hence, all subgroups).

### Trends in sodium intakes and blood pressure

Time trends of sodium intakes and measures of blood pressure over 8 years (between year 1 and year 9) were evaluated using adjusted linear regressions. Survey years were the unit of time used, as the distance between 2 surveys corresponds to an interval of a year. Time trends for sodium intakes were adjusted for age group, sex, BMI, ethnicity, equalized income, and daily energy intakes. Time trends for blood pressure were adjusted for the same factors, except that age was used as a continuous variable and the use of drugs affecting blood pressure was added as a binary covariate.

All analyses were carried out using R version 3.6.1 (R Foundation for Statistical Computing, Vienna, Austria).

## Results

### Changes in the density of foods consumed

The sodium density of all foods and beverages consumed by the UK population decreased by 17% between year 1 and year 9 of the NDNS (−16.1 mg/100 g; 95% CI: −20.4 to −11.8 mg/100 g; **Table 1**). In years 1 and 9, 2973 and 2897 distinct food products, respectively, were consumed (**Supplemental Table 1**). While the quantity of solid foods did not change, more drinks were consumed in year 9 than in year 1 (Supplemental Table 1). The decrease in the sodium density of solid foods only (i.e., beverages excluded) was of a lower intensity, but higher absolute value (−15%; −31.4 mg/100 g; 95% CI: −40.7 to −22.1 mg/100 g; Table 1). Most of the reduction in sodium density came from reformulation (−12.0 mg/100 g and −27.5 mg/100 g for all food and beverages and for solid food only, respectively). Product renewal had a smaller effect (−2.5 and −7.1 mg/100 g for all food and beverages and for solid food only, respectively) but still contributed to the decreased sodium density of foods consumed. In comparison, the effect of population switches between products was less clear. Switches between products (i.e., changes in quantities consumed) contributed to a decrease in sodium density of −1.6 mg/100 g when all food and beverages were considered, but they contributed to an increase in sodium density of 3.2 mg/100 g when only solid foods were considered. When table salt was excluded from the analysis, the effect of population switches between products resulted in a 9.3 mg decrease in the salt density of foods consumed (**Supplemental Table 2**). The proportion of participants declaring they added salt while cooking did not change over time (50% in year 1 and 54% in year 9; nonsignificant trend using a logistic regression).

**TABLE 1:** Sodium density of foods consumed from the NDNS survey, and changes in sodium density between Y1 and Y9

	All foods and beverages	Solid foods (beverages excluded)
Sodium density of foods, Y1	94.8 ± 1.5	213.6 ± 3.4
Sodium density of foods, Y9	78.7 ± 1.6	182.1 ± 3.3
Change in sodium density	−16.1 (−20.4 to −11.8)	−31.4 (−40.7 to −22.1)
% change in sodium density	−17% (−21% to −12%)	−15% (−19% to −10%)
Change in density from:		
Reformulation	−12.0	−27.5
Switches	−1.6	3.2
Product renewal	−2.5	−7.1

Data show population mean sodium densities of food consumed (in mg/100 g of foods) ± SEs of the means; absolute and percentage changes in sodium density between Y1 and Y9 (95% CI); and the effects of reformulation, switches, and product renewals on the changes in density (in mg/100 g), obtained from Equation 2. Population means are obtained from 1334 individuals in Y1 and 995 individuals in Y9. Abbreviation: Y, year.

The effects of reformulation, switches, and product renewals showed different patterns by category (**Table 2**). Reformulation had the highest effect in the cereals and cereal products category, leading to a decrease of 4.6 mg of sodium for 100 g of all foods consumed by the population (resulting from an average reformulation in this category of −48.7 mg/100 g; **Supplemental Table 3**). For this category, switches also contributed to the decrease in sodium density, with its effect being half of the effect of reformulation (−2.3 mg/100 g). Meat and meat products was the category with the second highest effect from reformulation (−3.0 mg/100 g), with an additional reduction of 2.8 mg/100 g from switches between products (and/or reducing the quantities eaten). While there was some reformulation in the sauces, soups, and condiments category (leading to a reduction in the sodium density of all foods by −1.8 mg/100 g), it was reduced by higher consumption of the items with high sodium in that category, especially table salt (leading to a 5.7 mg/100 g increase in the sodium density of foods consumed in a day). While beverages were not reformulated, their increased consumption led to a reduced sodium density of all foods consumed, as beverages have a lower sodium density than almost all other categories (the switches towards beverages led to a decrease in sodium density of −0.9 mg/100 g).

**TABLE 2:** Decomposition of the change in sodium density by food or beverage category

	Changes in sodium density, in mg/100 g of all foods and beverages consumed			Share of food category, % of total diet, in weight		
	Reformulation effect	Switching effect	Product renewal effect	Year 1	Year 9	% reformulation, in the food category
All foods and beverages	−12.0	−1.6	−2.5	—	—	—
Cereals & cereal products	−4.6	−2.3	−0.9	9%	9%	−15%
Meat and meat products	−3.0	−2.8	−1.2	6%	5%	−12%
Vegetables	−1.6	0.2	−0.9	10%	10%	−22%
Beverages	0.2	−2.2	0.3	52%	55%	+20%
Fish & fish products	−0.4	−0.6	0.0	1%	1%	−10%
Fats and oils	−0.2	−0.9	0.2	0%	0%	−10%
Eggs & egg dishes	−0.1	0.0	−0.2	1%	1%	−4%
Sugars, preserves & snacks	−0.3	−0.1	0.1	2%	2%	−8%
Nuts & seeds	0.0	−0.1	0.0	0%	0%	+2%
Supplements	0.0	0.0	0.0	0%	0%	−4%
Fruit	−0.3	0.9	−0.3	7%	6%	−47%
Milk & milk products	0.0	0.5	−0.1	10%	9%	0%
Sauces, soups and condiments	−1.8	5.7	0.5	2%	2%	−16%

Data in columns 1 to 3 show the effects of reformulation, switches, and product renewals, grouped by category (Equation 3). The changes in sodium density, grouped by category, indicate the contribution from each category to the total sodium density (i.e., for 100 g of all foods consumed, not for 100 g of foods from the category). Data in column 6 (percentage reformulation for products in the category) are the percentage changes in sodium density of foods in each category, relatively to the sodium density of the food categories in Y1 (Supplemental Equation*1*). There were 2973 foods in Y1 and 2897 in Y9 (breakout per category in Supplemental Table 1). Abbreviation: Y, year.

Reformulation, assessed at the level of the category (i.e., for 100 g of foods of this category), led to a decrease in sodium density in all categories except 2 (beverages and nuts and seeds; Supplemental Table 3). The intensity of reformulation was between 10%–20% in categories that contributed to a reduction in total intakes (Table 2).

The use of Equation 3 gave us a better understanding of the mechanisms behind the 3 effects (**Table 3**). There were only a few food products that had increased sodium density, as showed by the marginal effect of reformulations leading to increased sodium density. The overall small effect of switches between products was the result of 2 opposing effects: switches between categories were towards products with a lower sodium density, but switches between products within a same category were towards foods with higher sodium (−5.4 mg/100 g compared with +2.5 mg/100 g; Table 3). Most of the reduction in density from product renewal came from the delisting of products high in sodium, whereas new product introduction resulted in a small increase in sodium density.

**TABLE 3:** Further decomposition of the changes in sodium density of all foods and beverages consumed between Y1 and Y9, in mg/100 g

Sodium density, Y1	94.8 ± 1.5
Reformulation effect	−12.0
Reduction in sodium	−13.5
Increase in sodium	1.5
Switching effect	−1.6
Switches within categories^1^	2.5
Switches for reformulated products	1.2
Switches between categories^2^	−5.4
Product renewal effect	−2.5
Effect of product introduction	0.5
Effect of product delisting	−2.9
Sodium density, Y9	78.7 ± 1.6

Data were obtained using Equation 3. Population mean effects were obtained from 1334 individuals in Y1 and 995 individuals in Y9. Abbreviation: Y, year.

^1^Switches within categories result from different choices of products inside a category.

^2^Changes between categories show the overall effects of changes in shares of the different food categories, having different sodium densities.

### Stratification of changes by sociodemographic characteristics

**FIGURE 1:**
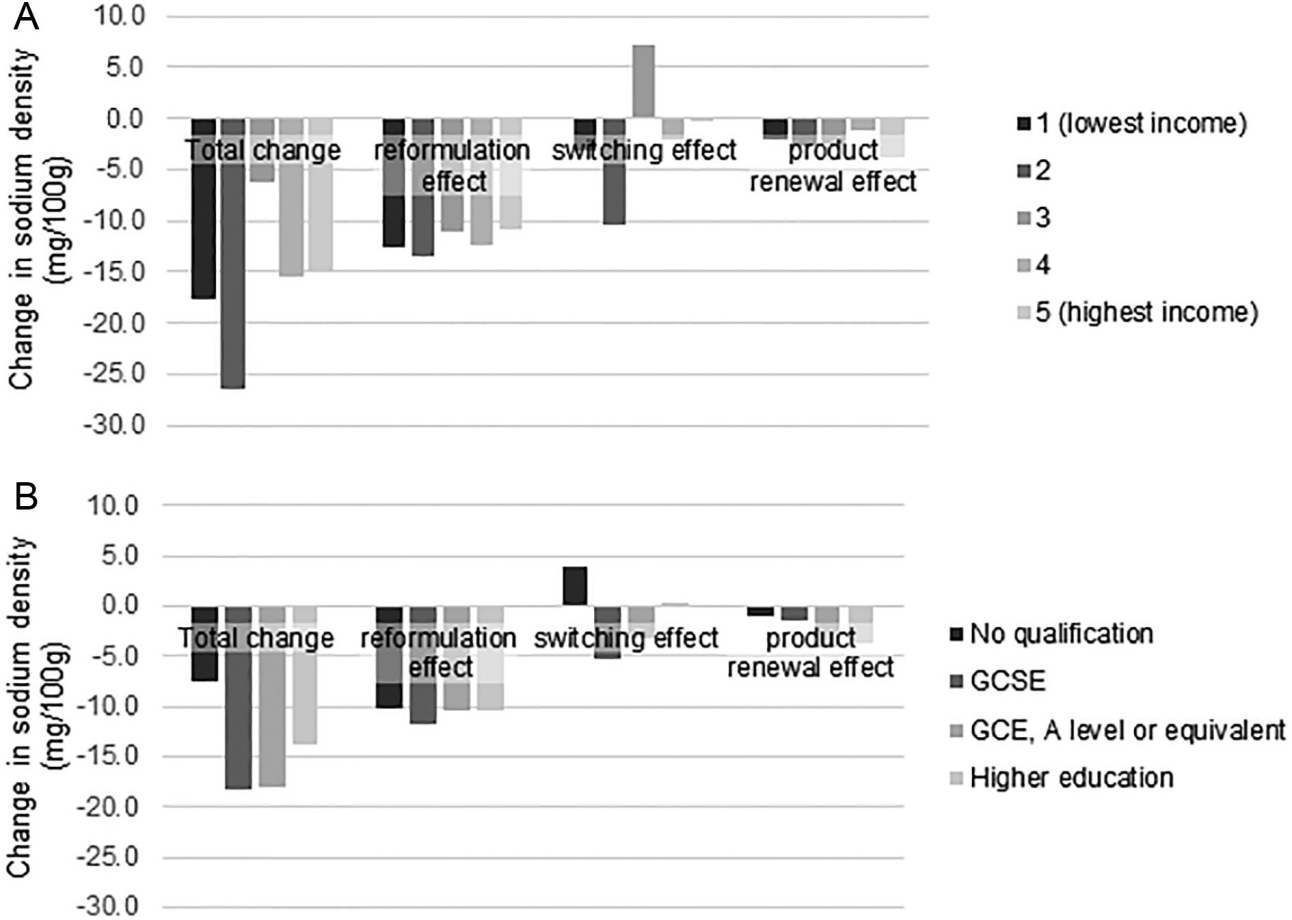
Changes in sodium density of all foods and beverages consumed between Y1 and Y9, by socioeconomic status. (A) The whole population (i.e., 1334 and 995 individuals in Y1 and Y9, respectively) was stratified by income quintile. (B) The adult population (i.e., 639 and 482 individuals in Y1 and Y9, respectively) was stratified by the highest qualification obtained. Abbreviations: GCSE, General Certificate of Secondary Education; GCE, General Certificate of Education; Y, year.

The stratification of the population by age, sex, or SES led to similar conclusions as for the whole population. For all stratifications, the dominant effect was the effect from reformulation, while the effects of product renewal and changes in food choices were smaller (**Supplemental Tables 4–7**). When the stratification was done by SES (either income or qualification), we observed that the effects of reformulation were consistent across groups, while the effects of switches showed disparities between groups, but no clear pattern between the direction and intensity of the change and the SES category (**Figure 1**).

### Trends in sodium intakes and blood pressure

**FIGURE 2:**
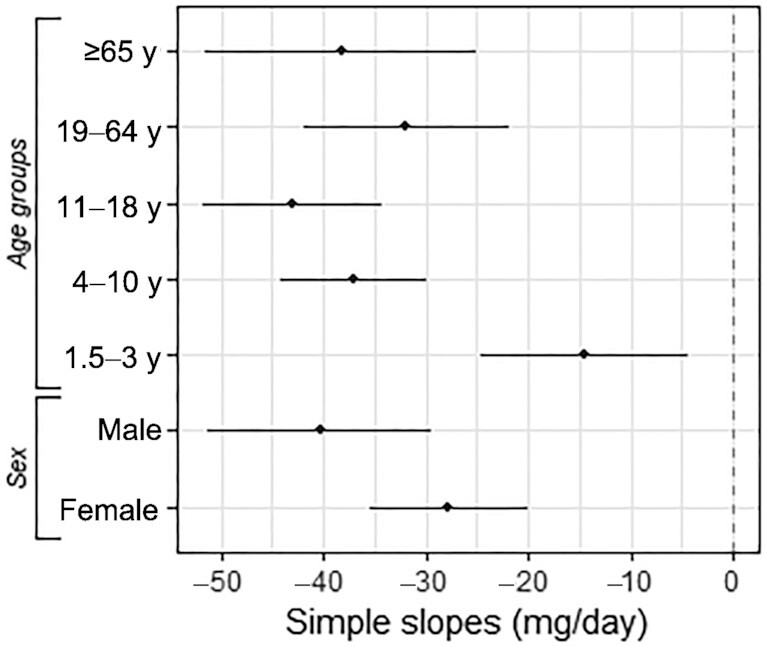
Estimated yearly changes in daily sodium intakes between 2008/09 and 2016/17. Simple slopes are in mg/d per survey year. Data show single slope estimates and SEs from adjusted time trends. All estimates have a *P* value < 0.05.

Between year 1 and year 9, participants had their daily sodium intakes decreased by 16% (95% CI: −21% to −12%; year 1: mean = 2293 mg/d, SEM = 43; year 9: mean = 1918 mg/d, SEM = 27). This reduction was observed across all age groups, and for both sexes (**Figure 2**).

Sodium intakes estimated using urine excretion also showed a decline between 2008/09 and 2012/13; however, this decline was not statistically significant. Sodium intakes measured using urine excretion were higher than those measured using food surveys, and the difference was higher for high-sodium consumers (**Supplemental Figure 4**). Systolic blood pressure also showed a decreasing trend between year 1 and year 9, after adjusting for confounders. The estimated reduction was −0.4 mmHg (SEM, 0.1; *P* value < 0.0001) per survey year.

## Discussion

This study showed that the 16% decrease in sodium intakes recorded between 2008/09 and 2016/17 was accompanied by a 17% decrease in the sodium density of foods and beverages consumed. There was a 15% decrease in the sodium density of solid foods, with no change in the quantity consumed. This decrease in sodium density was mostly explained by the reformulation of food products, while the effects of product renewal and changes in food choices had only small contributions. Similar results were found when stratifying for age, sex, or SES. Reformulation changes the food environment people live in, and this study suggests that changing the food environment has a higher impact on improving diets than changes in consumer choices. Several systematic reviews on strategies to improve diets also found that reformulation reduced sodium intakes (16, 17). In addition, a study on the nutritional quality of French purchases in 4 categories using a similar methodology found a stronger improvement due to reformulation, while the effects of product renewal and changes in food choices did not have such impacts (18).

The sodium density of foods was only slightly changed by population switches between products. This resulted from the balance between choices of products with less sodium between categories, but adverse choices within categories (Table 3). Better choices were made within the categories of cereals and cereal products and meat and meat products. The reduction of the quantities of meat products consumed, and the increased consumption of beverages, explained the beneficial effect observed from switches between categories. Better food choices can be the result of public health campaigns aiming at helping consumers making healthier choices within categories (using salt labeling, for example) and between categories (for example, campaigns highlighting the high sodium content of some processed meat). The fact that our 2 estimates of sodium intakes (from food diaries and urinary excretion) showed a decrease in sodium intakes suggests that people did not (or only partially) compensate for the reduced sodium in food products by adding salt while eating. However, this finding needs to be taken cautiously, as the NDNS was not set to capture use of table salt.

In this study, we found that reformulation was the driver of the reduction in sodium density of foods for all subgroups defined by their SES, and its effect was of a similar intensity for all groups. As such, reformulation is not expected to have an impact on existing disparities. The study by Griffith et al. (9) found a higher impact of reformulation on most deprived households, but this was offset by switches between products. A microsimulation study modeling the longer-term impacts of the salt reduction observed in the United Kingdom until 2011 found no reduction in health disparities (19). In a study evaluating the impact of the early years of the UK salt reduction program on salt intakes, all income, age, and sex groups had decreased intakes of sodium, with a stronger decrease in women than in men (20). However, there are concerns that policies based on education could have higher effects on educated populations, and hence would widen disparities, as higher-income populations tend to have better diets than lower-income populations (20, 21).

Not all categories contributed equally to the decrease in salt density. This was expected, as the salt reduction program targets priority food categories that are the main contributors to sodium in the diet, such as bread, meat-based products, soups, or sauces (22). We found that these categories have indeed been reformulated, with sodium reductions of about 10%–20%. A previous study using UK purchases found that dairy, convenience foods, snacks, sauces, and cereal products were reformulated by 10%–30% between 2006 and 2011 (7). Public Health England (PHE) tracks the proportion of products reaching the reformulation targets set in the salt reduction program. In their 2018 report, they found that breakfast cereals, breads, stocks and gravies, and soups had more than 75% of their products reaching the targets, while only 50%–60% of sauces, canned vegetables, or meat products met the target (22). The difference between the PHE study and results from this study suggests that while some high-sodium categories were reformulated, they are still far from the targets set.

In this study, we found a 16% reduction in dietary salt intakes between 2008/09 and 2016/2017, estimated with food diaries. These changes were smaller than the unadjusted trends reported in the NDNS report (23). This difference could be explained by the adjustments for sociodemographic characteristics and energy intakes we have made. In comparison, we found a higher reduction than the UK salt survey, which found no change in (unadjusted) salt intakes between 2008/09 and 2018/09 (after a decrease in salt intakes from 2005/06 to 2008/09) (13). The UK salt survey used 24-h urinary excretion, a measure that covers all salt intakes, in contrast to diet surveys, in which participants may omit some foods eaten. In our sensitivity analysis using the 24-h urine excretion data from the NDNS, we found a small but nonsignificant decrease between 2008/09 and 2012/13 after adjusting for demographic factors and energy intakes. We also found a decrease in systolic blood pressure of −0.4 ± 0.1 mmHg per survey year. Although we adjusted for BMI and drug use, we could not assume a causal relationship between the observed decreases in salt intakes and in blood pressure. A study on the UK population also found a reduction in blood pressure concurrent to a decrease in salt intakes between 2003 and 2011 (24). This study controlled for more confounders than our study and attributed the decrease in blood pressure to changes in salt intakes, suggesting this link could exist in our study.

To continue the reduction in salt intakes of the UK population, new salt reduction guidelines were published in 2020 (25). In the latest salt survey of 2018/2019, the mean salt intake was 40% higher than the recommendation of 6 g/d (13), stressing the need for manufacturers to continue their reformulation efforts and for the population to adopt healthier behaviors.

### Strengths and limitations

To our knowledge, this is the first study where the decomposition method has been applied to a diet survey. Previously, this method has been applied to food purchase data from household panels (9, 18). Applying this method to the NDNS allowed us to understand how the sodium density of all foods consumed changed over time, while purchase data from household panels normally only record foods purchased for home consumption and for the whole household. Also, the estimations we obtained from the NDNS are representative of the UK population (and subgroups of populations). Although the cross-sectional design of the NDNS prevented the use of rigorous causal inference approaches, we found that the decrease in sodium density was accompanied by a decrease in sodium intakes (estimated using food diaries and urine excretion), as well as a decrease in blood pressure.

However, applying this method on the NDNS generic food composition table implied that we measured changes in supply (reformulation and product renewal) at a level that is not granular enough to get the effect of reformulation and product renewal happening at the branded product level (26). Applying the decomposition method on aggregated food intake data may miss switches between similar products or different reformulation strategies of manufacturers of similar products. Furthermore, because population intakes were estimated from a sample of the population, product renewal may have been overestimated, and may represent switches between products still available on the market. As the NDNS food codes gather products that are similar, the differences in their sodium contents should be small. In addition, timing could be biased, as nutrition information used in the NDNS (from the UK Nutrient Databank) is not updated every year. The databank is updated regularly; however, this process takes time, so may create a lag between the actual composition of food products and their nutrition composition in the NDNS. However, our estimates using a lagged nutrition composition should underestimate, and not overestimate, the effects of reformulation. A study done in France showed that when estimating dietary intakes from branded foods only, retailer brands only or a full representation of products matching a generic description led to similar dietary intakes (27), suggesting that what we observe using generic food codes should be similar to what we would observe with more granular data. Hence, we are confident that our estimates are close to what would have been obtained using more detailed information on food intakes and food composition.

Another limitation of our study is linked to the method the NDNS used for recording salt added while cooking or eating. Participants were not asked directly to write in their diaries whether they were using table salt. Some participants voluntarily declared it, while other did not (23). First, it means that the sodium density we estimated is lower than the actual sodium density of all food and beverage intakes. As we do not expect a change in the declaration of table salt over time, the irregular declaration of table salt should not be a problem for our study, which focused on differences over time. Also, table salt was estimated to represent 15% of sodium consumed in the United Kingdom, while 80% was from manufactured foods (28). In this study, we used sodium intakes measured from food surveys, a method known to be less accurate than 24-h urine collection: there was a risk of misreporting of salt intakes. However, the methodologies to correct for misreporting can create bias themselves. Although there may be a declaration bias, the proportion of participants of the NDNS who reported adding salt when cooking did not change between year 1 and year 9. In addition, we also measured a downward trend in sodium intakes estimated using urinary excretion, suggesting that actual total sodium intakes decreased over time. Second, there could be a bias in the estimation of sodium intakes if those participants declaring use of table salt were different from those participants omitting such a declaration. Our sensitivity analysis excluding table salt found similar results to our main analysis, supporting the validity of our results.

### Conclusion

The sodium density of foods and beverages consumed by the UK population decreased by 17% between 2008/2009 and 2016/2017, associated with a 16% decrease in sodium intakes. This reduction was largely driven by reduction in the sodium content of foods (reformulation). Changes in food choices indicated a trend toward lower-salt products, except for in the condiment category, balancing healthier choices in other categories. Similar results were found across demographic and socioeconomic groups, suggesting a homogenous effect of the UK salt reduction program on the population.

## Supplementary Material

nqab130_Supplemental_File

## Data Availability

Data used in the study was obtained from the UK Data Service. Data described in the manuscript and code book will be made available upon request pending application and approval to the UK Data Service. The analytic code will be made available upon request pending application and approval to the corresponding author.

## Notes

MG is the recipient of an unconditional PhD Studentship grant from Societé des Produits Nestlé, supervised by GF. Societé des Produits Nestlé had no role in the study design, data collection and analysis, or preparation of the protocol and research paper. (Science and Technology in childhood Obesity Policy) project. The STOP project (http://www.stopchildobesity.eu/) received funding from the European Union's Horizon 2020 research and innovation program under Grant Agreement No. 774548. The Nutrition Research Group is funded by grants from the Medical Research Council, the Biotechnology and Biological Sciences Research Council, the National Institute for Health Research (NIHR); an Integrative Mammalian Biology Capacity Building Award; and the NIHR Biomedical Research Centre Funding Scheme.

The content of this publication reflects only the views of the authors, and the European Commission is not liable for any use that may be made of the information it contains. The views expressed are those of the author(s) and not necessarily those of the National Health Service, the National Institute for Health Research, or the Department of Health.

Supplemental Tables 1–7, Supplemental Figures 1–4, and Supplemental Equation *1* are available from the “Supplementary data” link in the online posting of the article at https://academic.oup.com/ajcn/.

Abbreviations used: NDNS, National Diet and Nutrition Survey; PHE, Public Health England; SES, socioeconomic status.
