# Supplementary material for: Contribution of reformulation, product renewal, and changes in consumer behavior to the reduction of salt intakes in the UK population between 2008/2009 and 2016/2017
Source: Am J Clin Nutr. 2021 May 8;114(3):1092–9. doi: 10.1093/ajcn/nqab130 (PMC8408870; doi:10.1093/ajcn/nqab130)
Supplement: nqab130_Supplemental_File [file nqab130_supplemental_file.docx]

# On-line Supplementary Material

Contribution of reformulation, product renewal and changes in consumer behavior to the reduction of salt intakes in the UK population between 2008/2009 and 2016/2017

Authors: Mathilde Gressier, Franco Sassi, Gary Frost;

Correspondence to: [m.gressier18@imperial.ac.uk](mailto:m.gressier18@imperial.ac.uk)

## **Supplementary Equations**

$$Reformulation in the category k=\sum_{i\in k\cap C} w_{i,t_{0}}^{k}\left( n_{i,t_{1}}-n_{i,t_{0}} \right)$$

$with w_{i,t_{0}}^{k}=\frac{weight food i}{weight all foods in k}$

Supplementary equation 1: Reformulation calculated at the food category level.

## **Supplementary Figures**


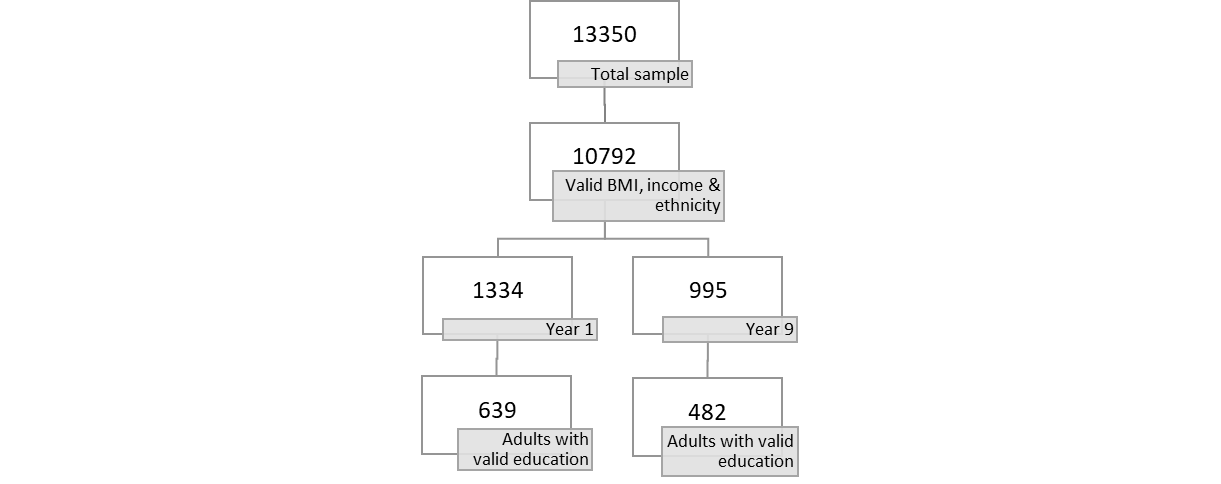


Supplementary figure 1: Flow chart of the selection of NDNS participants with valid food diary data for this study. All participants in the total sample had valid food diaries. Participants with valid BMI, income and ethnicity were used to derive average population dietary intakes. Data in year 1 and year 9 were used for the decomposition analysis. Only adults were included in the stratification by education


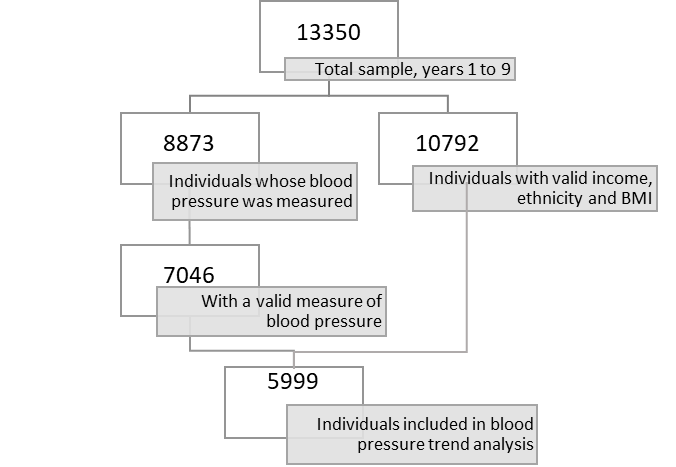


Supplementary figure 2: Flow chart of the selection of NDNS participants with valid blood pressure data, included in trend analyses


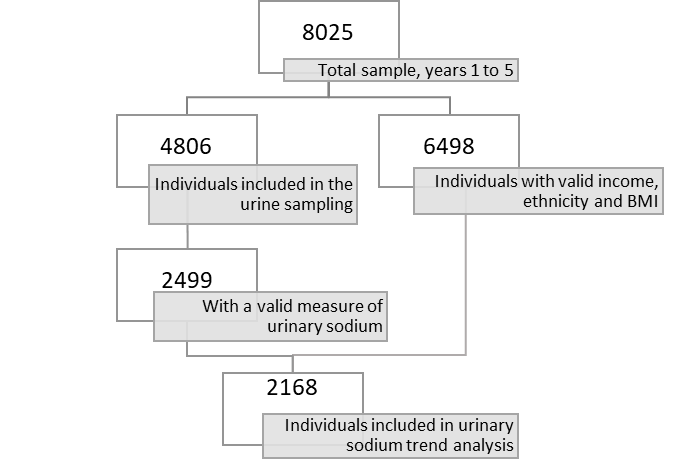


Supplementary figure 3: Flow chart of the selection of NDNS participants with valid measure of urinary sodium, included in trend analyses (from years 1 to 5)


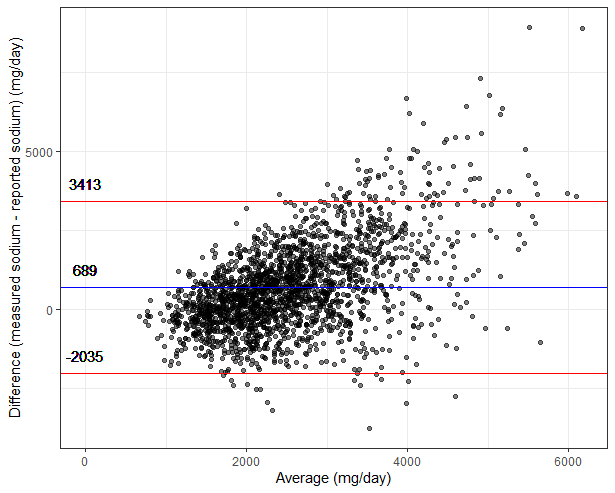


Supplementary figure 4: Bland-Altman plot comparing the two estimations of sodium intakes: measured with 24h-urine excretion and reported from food diaries, for the 2168 participants with valid 24-h collection and demographic characteristics (NDNS years 1 to 5).

The blue line represents the mean difference, the red lines the confidence interval around the mean difference.

## **Supplementary Tables**

Supplementary table 1: Number of products, percentage of participants consuming products from food categories, and mean quantity consumed in Year 1 and Year 9.

|  | **N products** | | **Percentage of consumers**  **(% ± SEM)** | | | **Mean quantity consumed**  **(g/day ± SEM)** | | |  |
| --- | --- | --- | --- | --- | --- | --- | --- | --- | --- |
|  | Year 1 | Year 9 | Year 1 | Year 9 | p-val trend^1^ | Year 1 | Year 9 | p-val trend^1^ | |
| Beverages | 192 | 188 | 100% ±0% | 100% ±0% | *0.0203* | 1452 ±31 | 1521 ±37 | *3.627E-05* | |
| Cereals & cereal products | 665 | 635 | 100% ±0% | 100% ±0% | *0.0152* | 222 ±4 | 215 ±5 | 0.5074 | |
| Eggs | 59 | 44 | 54% ±2% | 65% ±2% | *3.75E-07* | 16 ±1 | 24 ±1 | *0.0030* | |
| Fats and oils | 46 | 54 | 86% ±1% | 94% ±1% | *5.56E-10* | 9 ±0 | 9 ±0 | 0.3875 | |
| Fish & fish products | 140 | 113 | 60% ±2% | 60% ±2% | 0.6460 | 29 ±1 | 25 ±1 | 0.5841 | |
| Fruit | 147 | 184 | 88% ±1% | 86% ±1% | 0.1863 | 158 ±6 | 153 ±8 | 0.1570 | |
| Meat and meat products | 481 | 396 | 97% ±1% | 93% ±2% | *0.0137* | 139 ±3 | 115 ±5 | *0.0010* | |
| Milk & milk products | 205 | 228 | 98% ±1% | 99% ±1% | 0.7929 | 222 ±6 | 221 ±7 | 0.3609 | |
| Nuts & seeds | 37 | 39 | 21% ±1% | 37% ±2% | *7.34E-20* | 3 ±0 | 9 ±2 | *4.741E-05* | |
| sauces, soups and condiments | 238 | 233 | 93% ±1% | 98% ±0% | *3.76E-06* | 52 ±2 | 44 ±3 | *0.0112* | |
| Sugars, preserves & snacks | 181 | 180 | 92% ±1% | 95% ±1% | *0.0004* | 38 ±1 | 36 ±1 | 0.3692 | |
| Supplements | 197 | 206 | 31% ±2% | 31% ±2% | 0.4418 | 1 ±0 | 1 ±0 | 0.2861 | |
| Vegetables | 385 | 397 | 100% ±0% | 99% ±0% | 0.1704 | 257 ±5 | 268 ±7 | *0.0203* | |
| Solid foods | 2751 | 2677 |  |  |  | 1088 ±13 | 1070 ±15 | 0.4167 | |
| Drinks^2^ | 222 | 220 |  |  |  | 1517 ±32 | 1572 ±38 | *3.111E-05* | |

^1^p-values for trend were calculated using linear regression for all survey years from year 1 to year 9, adjusted for sex, age group, ethnicity, equivalised income, energy intakes and BMI.

^2^ Drinks: beverages and fruit juices

Supplementary table 2: Sodium density of foods consumed and changes in sodium density between Y1 and Y9 of NDNS survey.

|  | **all foods and drinks**  **Y1: n products=2973**  **Y9: n products=2897** | **no drinks**  **Y1: n products=2754**  **Y9: n products=2677** | **no drinks, no table salt**  **Y1: n products=2753**  **Y9: n products=2676** |
| --- | --- | --- | --- |
| Sodium density of foods, Year 1 | 94.8 ± 1.5 | 213.6 ± 3.4 | 212.0 ± 3.4 |
| Sodium density of foods, Year 9 | 78.7 ± 1.6 | 182.1 ± 3.3 | 168.2 ± 3.2 |
| Change in sodium density | -16.1 (-20.4, -11.8) | -31.4 (-40.7, -22.1) | -43.9 (-53.1, -34.7) |
| *% change in sodium density* | -17% (-21%, -12%) | -15% (-19%, -10%) | -21% (-25%, -16%) |
| Change in density by: |  |  |  |
| reformulation effect | -12.0 | -27.5 | -27.5 |
| switching effect | -1.6 | 3.2 | -9.3 |
| product renewal effect | -2.5 | -7.1 | -7.1 |

Data shows mean sodium densities of food consumed (in mg/100g of foods) ± standard error of the means. The 95% confidence interval of the absolute and percentage change is indicated in parentheses.

Supplementary table 3: Sodium density by food category in Y1 and Y9, average reformulation of the category, and shares in weight of the category in the total diet. Y1: n=2973 foods and beverages, Y9: n=2897 foods and beverages

|  | Average sodium density of the category (mg/100g)^1^ | | Average reformulation^2^ | | Shares of the category (%, in weight) | |
| --- | --- | --- | --- | --- | --- | --- |
|  | Y1 | Y9 | (mg/100g) | % reduction | Y1 | Y9 |
| Cereals & cereal products | 318.7 | 240.3 | -48.7 | *-15%* | 9% | 9% |
| Milk & milk products | 92.1 | 95.3 | -0.2 | *0%* | 9% | 9% |
| Eggs | 250.0 | 170.9 | -10.4 | *-4%* | 1% | 1% |
| Fats and oils | 518.3 | 305.0 | -52.6 | *-10%* | 0% | 0% |
| Meat and meat products | 428.1 | 349.4 | -53.1 | *-12%* | 6% | 2% |
| Fish & fish products | 347.4 | 303.2 | -33.4 | *-10%* | 1% | 1% |
| Vegetables | 72.1 | 50.4 | -15.6 | *-22%* | 10% | 10% |
| Fruit | 11.0 | 8.1 | -5.1 | *-47%* | 7% | 6% |
| Sugars, preserves & snacks | 196.1 | 189.0 | -15.2 | *-8%* | 2% | 2% |
| Beverages | 2.2 | 2.9 | 0.4 | *20%* | 52% | 55% |
| sauces, soups and condiments | 525.0 | 851.9 | -84.2 | *-16%* | 2% | 2% |
| Supplements | 30.5 | 22.3 | -1.2 | *-4%* | 0% | 0% |
| Nuts & seeds | 170.5 | 97.1 | 3.8 | *2%* | 0.% | 0% |

^1^: the sodium density is calculated for 100g of products in this category, by rescaling the shares of each food product such as the sum of shares per category equals 100%.

^2^: reformulation calculated with equation 4 above. It does not correspond to the difference in sodium density between Y1 and Y9 as changes there were changes in the shares of products inside each category.

Legend of supplementary tables 4-7: Sodium density of foods consumed (all foods and beverages) and changes in sodium density between Y1 and Y9 of NDNS survey, stratified by age, gender, or socio-economic status. Data shows mean sodium densities of food consumed (in mg/100g of foods). The 95% confidence interval of the percentage change is indicated in parentheses.

Supplementary table 4:stratification by age group

| Age group | **1.5-3y**  Y1: n=106  Y9: n=75 | **4-10y**  Y1: n=292  Y9: n=221 | **11-18y**  Y1: n=283  Y9: n=197 | **19-64y**  Y1: n=526  Y9: n=391 | **>65y**  Y1: n=127  Y9: n=111 |
| --- | --- | --- | --- | --- | --- |
| Sodium density of foods, Year 1 | 97.6 ± 4.3 | 121.5 ± 2.6 | 121.7 ± 3 | 89.6 ± 2.1 | 82.6 ± 3.4 |
| Sodium density of foods, Year 9 | 93.1 ± 3.7 | 101 ± 2.4 | 98.4 ± 3.3 | 74.4 ± 1.9 | 67.8 ± 3.7 |
| Change in sodium density | -4.5 | -20.5 | -23.3 | -15.2 | -14.7 |
| *% change in sodium density* | -5%  (-16%,7%) | -17%  (-23%,-11%) | -19%  (-26%,-12%) | -17%  (-23%,-11%) | -18%  (-30%,-6%) |
| reformulation effect | -13.5 | -17.8 | -16.9 | -11.1 | -9.1 |
| switching effect | 6.5 | 0.4 | -5.2 | -1.3 | -2.8 |
| product renewal effect | 2.5 | -3.0 | -1.1 | -2.7 | -2.9 |

Supplementary table 5: Stratification by sex

|  | **Males**  Y1: n=621  Y9: n=476 | **Females**  Y1: n=713  Y9: n=519 |
| --- | --- | --- |
| Sodium density of foods, Year 1 | 99.2 ± 2.3 | 90.6 ± 1.9 |
| Sodium density of foods, Year 9 | 82.6 ± 2.3 | 74.7 ± 1.9 |
| Change in sodium density | -16.7 | -15.9 |
| *% change in sodium density* | -17%  (-23%,-10%) | -18%  (-23%,-12%) |
| reformulation effect | -12.8 | -11.3 |
| switching effect | -1.1 | -2.4 |
| product renewal effect | -2.8 | -2.2 |

supplementary table 6: stratification by qualification

| Highest qualification obtained | **No qualification**  Y1: n=164  Y9: n=92 | **GCSE**  Y1: n=148  Y9: n=106 | **GCE, A level or equivalent**  Y1: n=114  Y9: n=78 | **Higher education**  Y1: n=213  Y9: n=206 |
| --- | --- | --- | --- | --- |
| Sodium density of foods, Year 1 | 85.6 ± 2.6 | 93.2 ± 3.7 | 90.7 ± 4.3 | 85.4 ± 3 |
| Sodium density of foods, Year 9 | 78.1 ± 5 | 74.9 ± 4.4 | 72.7 ± 3.4 | 71.5 ± 2.9 |
| reduction in mg/100g | -7.5 | -18.3 | -18.0 | -13.9 |
| *% change in sodium density* | -9%  (-22%,4%) | -20%  (-32%,-8%) | -20%  (-32%,-8%) | -16%  (-26%,-7%) |
| reformulation effect | -10.3 | -11.7 | -10.5 | -10.5 |
| switching effect | 3.8 | -5.2 | -3.3 | 0.3 |
| product renewal effect | -1.0 | -1.4 | -4.2 | -3.7 |

Supplementary table 7: Stratification by income

| Quintile of income | **1 (lowest income)**  Y1: n=312  Y9: n=251 | **2**  Y1: n=246  Y9: n=186 | **3**  Y1: n=295  Y9: n=211 | **4**  Y1: n=223  Y9: n=201 | **5 (highest income)**  Y1: n=258  Y9: n=146 |
| --- | --- | --- | --- | --- | --- |
| Sodium density of foods, Year 1 | 97.3 ± 3.1 | 102.1 ± 3.9 | 91.8 ± 2.6 | 94.7 ± 3.3 | 88.3 ± 2.6 |
| Sodium density of foods, Year 9 | 79.6 ± 2.4 | 75.6 ± 3.9 | 85.5 ± 3.8 | 79.2 ± 3.4 | 73.2 ± 3.3 |
| Change in sodium density | -17.7 | -26.5 | -6.3 | -15.5 | -15.0 |
| *% change in sodium density* | -18%  (-26%,-10%) | -26%  (-37%,-15%) | -7%  (-17%,3%) | -16%  (-26%,-6%) | -17%  (-26%,-8%) |
| reformulation effect | -12.6 | -13.5 | -11.0 | -12.3 | -10.9 |
| switching effect | -3.1 | -10.3 | 7.3 | -2.0 | -0.3 |
| product renewal effect | -2.0 | -2.7 | -2.5 | -1.1 | -3.9 |
